# Supplementary material for: Reduced C9orf72 function leads to defective synaptic vesicle release and neuromuscular dysfunction in zebrafish
Source: Commun Biol. 2021 Jun 25;4:792. doi: 10.1038/s42003-021-02302-y (PMC8233344; doi:10.1038/s42003-021-02302-y)
Supplement: Supplementary file 2 — Supplementary Information [file 42003_2021_2302_MOESM2_ESM.pdf]

Supplementary Information

Reduced C9orf72 function leads to defective synaptic vesicle release and neuromuscular dysfunction in zebrafish

Zoé Butti[1], Yingzhou Edward Pan[1], Jean Giacomotto[2,3], Shunmoogum A. Patten[1,4*]

***Correspondence:** Correspondence should be addressed to

Shunmoogum (Kessen) Patten
INRS- Centre Armand-Frappier Santé Biotechnologie
531 Boulevard des Prairies
Laval, Quebec
H7V 1B7
Canada
kessen.patten@inrs.ca

Affiliations:
1. INRS- Centre Armand-Frappier Santé Biotechnologie, Laval, QC, Canada.
2. Queensland Brain Institute, University of Queensland, St Lucia, Queensland, Australia
3. Queensland Centre for Mental Health Research, Brisbane, Queensland, Australia
4. Centre d'Excellence en Recherche sur les Maladies Orphelines - Fondation Courtois (CERMO-FC), Université du Québec à Montréal (UQAM), Montréal, QC, Canada.

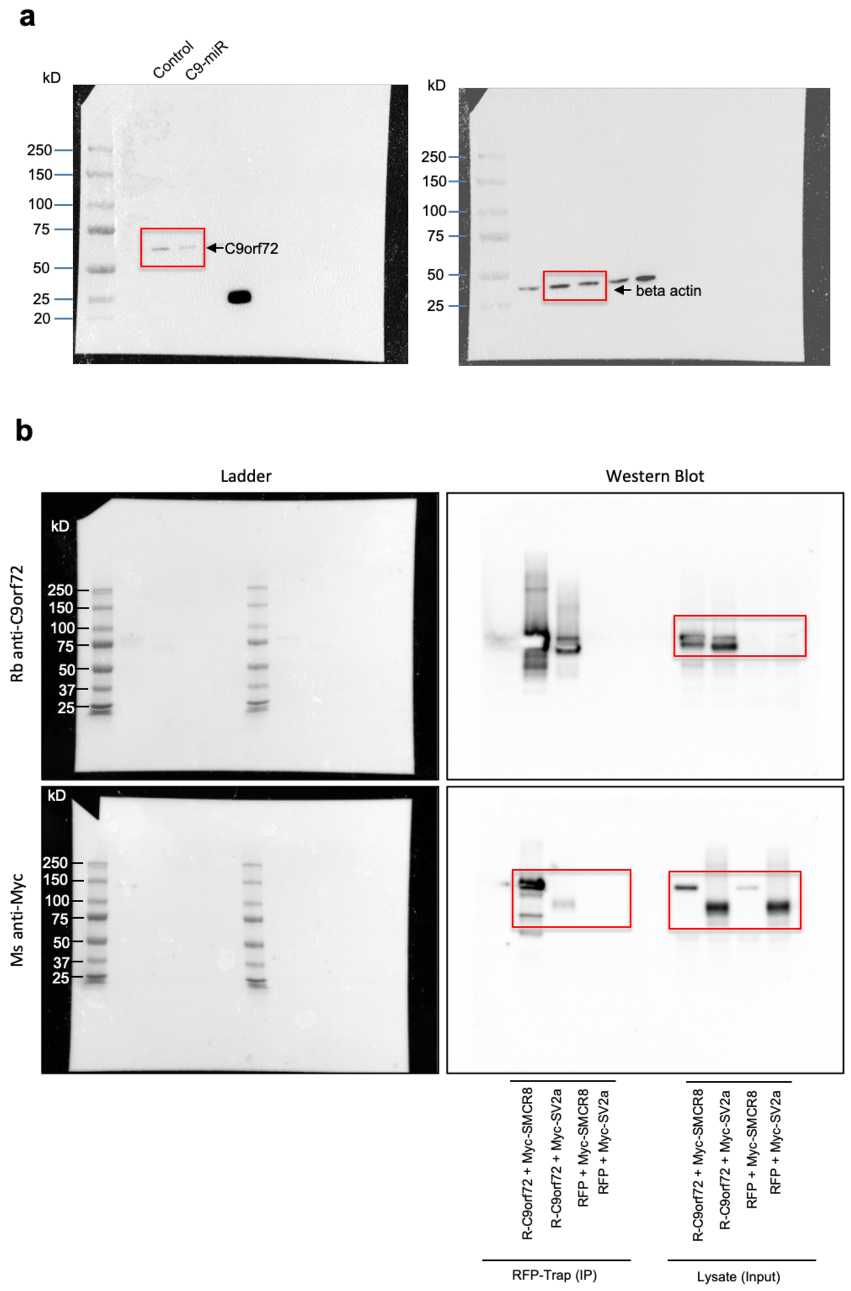

Supplementary Figure 1: Full Western blot for (a) Figure 1d and (b) Figure 7c.

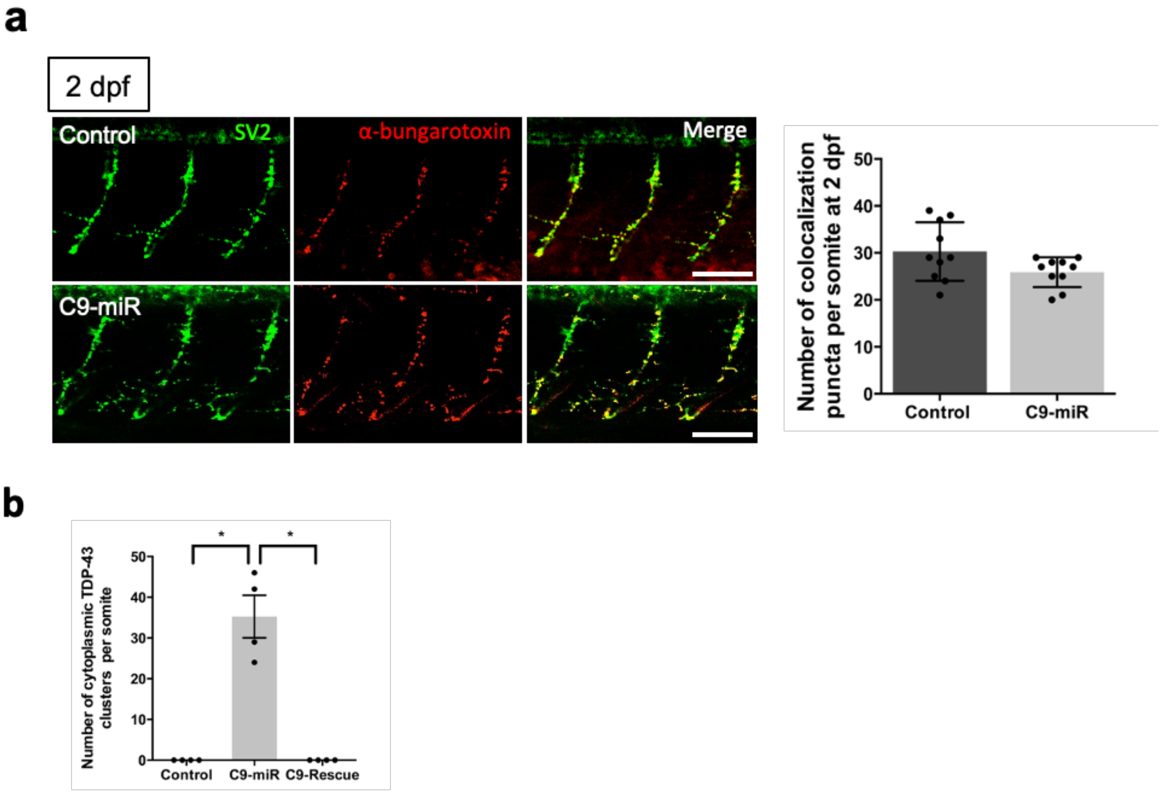

Supplementary Figure 2: Characterization of C9-miR fish.

a. Representative images of co-immunostaining of zebrafish neuromuscular junctions with presynaptic (SV2) and postsynaptic (α-bungarotoxin) markers in 2 dpf zebrafish. Scale bar = 50 μm. Quantification of the colocalizing pre-and post-synaptic markers per somite showed no differences between C9-miR and controls at early embryonic stages (2 dpf; n=10; p=0.064; Student's t-test). **b.** Quantification of cytoplasmic TDP-43 clusters in control, C9-miR and C9-rescue fish (n=4; *p<0.05; One-way ANOVA). Data are presented as mean±SEM. n represents number of fish.

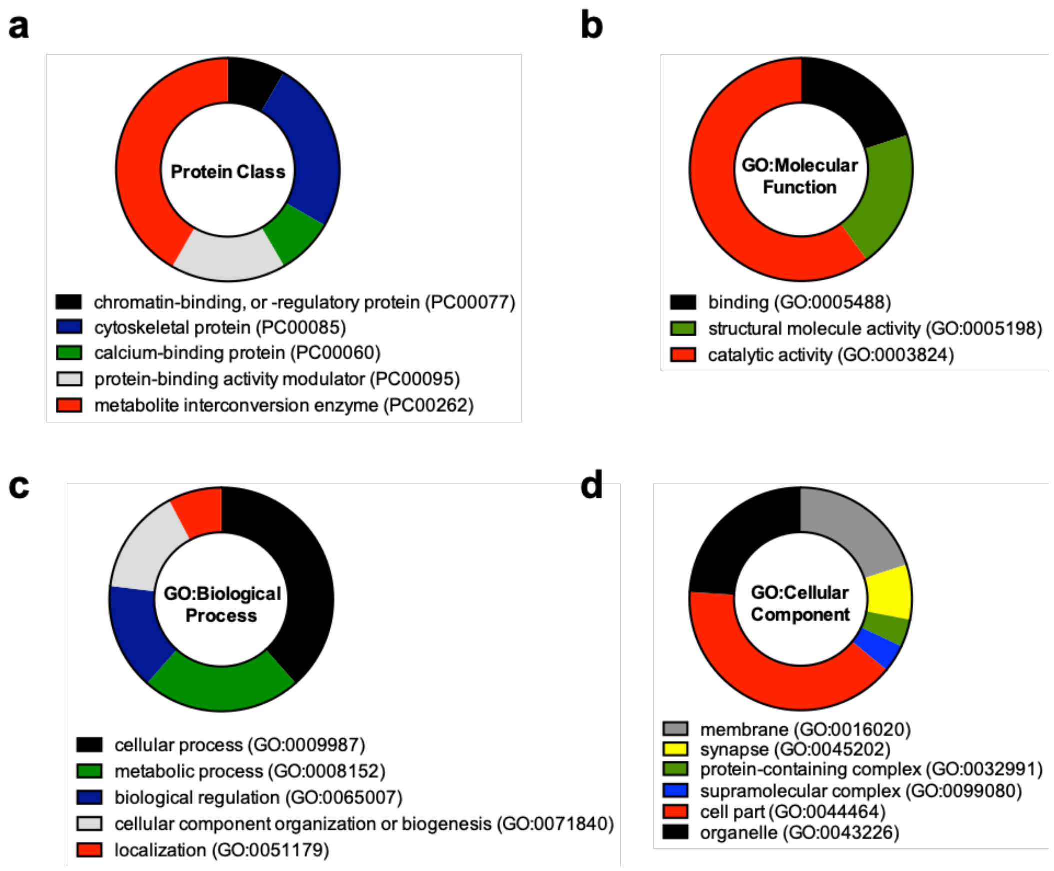

Supplementary Figure 3: Proteomic analysis in C9-miR and control larvae.

Protein Class (**a**), Molecular function (**b**), Biological processes (**c**) and Cell component (**d**) GO-term classifications that are enriched in the differentially expressed proteins.

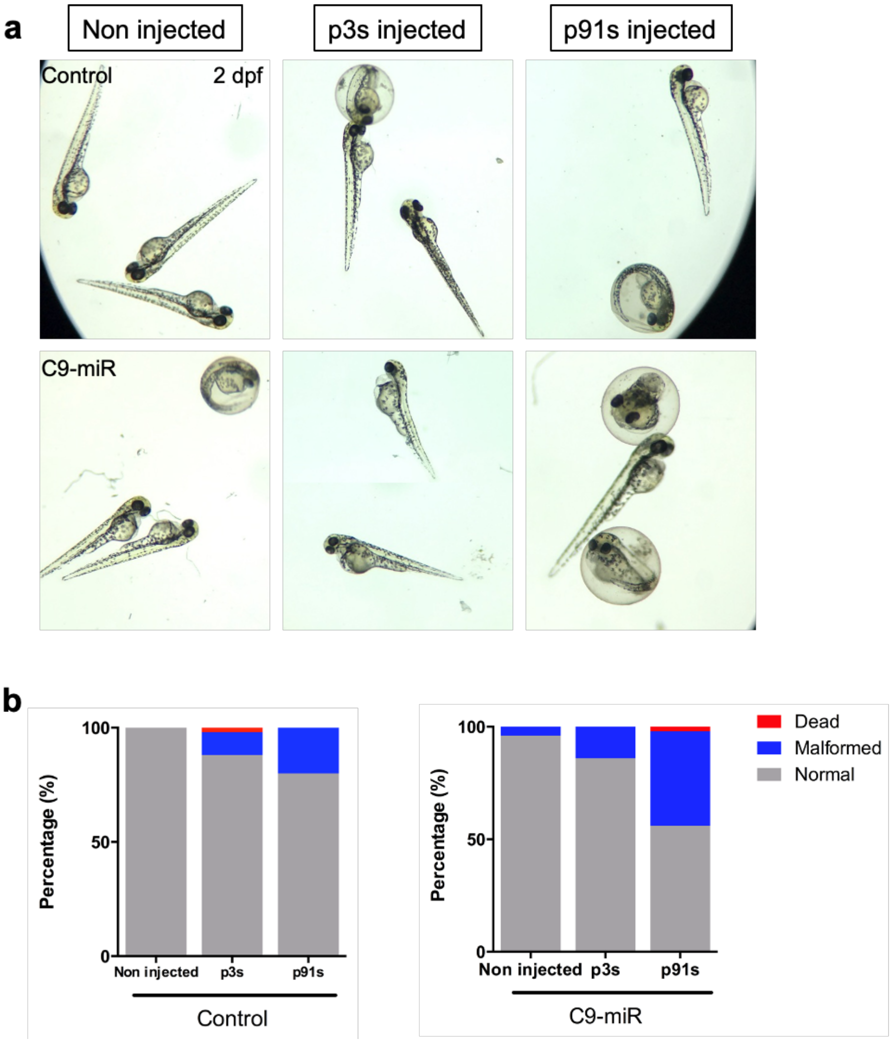

Supplementary Figure 4: Expression of long GGGGCC repeats exacerbates toxicity in C9-miR fish.

a. Images of the control and C9-miR zebrafish 2dpf fish uninjected or injected with GGGGCC short (p3s) and long (p91s) mRNA. **b.** Expressing p91s mRNA in C9-miR increases toxicity (*right panel*; Chi-square=53.72, df=4, N=3, n=75) compared to control (*left panel*). N represents number of experimental repeats and n represents number of fish.

Supplementary Table 1 - Design of 4x anti-c9orf072 miRNAs stem loops compatible with the pME-RNAi642

NAME	SEQUENCE
C9ORF72-01 mature miR (5'-3')	ACATCAAGACGCAAGAGTGTG
C9ORF72-02 mature miR (5'-3')	TGCAGAATGCTGTAAACAAAG
C9ORF72-03 mature miR (5'-3')	TTCATAAACCTCAAGACCACA
C9ORF72-04 mature miR (5'-3')	CTATTAGTCAACAAGCCGTTA
C9ORF72-01 star miR (5'-3')	CACACTCTCGTCTTGATGT
C9ORF72-02 star miR (5'-3')	CTTTGTTTAGCATTCTGCA
C9ORF72-03 star miR (5'-3')	TGTGGTCTAGGTTTATGAA
C9ORF72-04 star miR (5'-3')	TAACGGCTTTGACTAATAG
C9ORF72-01a stem loop	TGCTGACATCAAGACGCAAGAGTGTGGTTTTGGCCACTGACTGACCACACTCTCGTCTTGATGT
C9ORF72-01b stem loop	CCTGACATCAAGACGAGAGTGTGGTCAGTCAGTGGCCAAAACCACACTCTTGCGTCTTGATGTC
C9ORF72-02a stem loop	TGCTGTGCAGAATGCTGTAAACAAAGGTTTTGGCCACTGACTGACCTTTGTTTAGCATTCTGCA
C9ORF72-02b stem loop	CCTGTGCAGAATGCTAAACAAAGGTCAGTCAGTGGCCAAAACCTTTGTTTACAGCATTCTGCAC
C9ORF72-03a stem loop	TGCTGTTCATAAACCTCAAGACCACAGTTTTGGCCACTGACTGACTGTGGTCTAGGTTTATGAA
C9ORF72-03b stem loop	CCTGTTCATAAACCTAGACCACAGTCAGTCAGTGGCCAAAACTGTGGTCTTGAGGTTTATGAAC
C9ORF72-04a stem loop	TGCTGCTATTAGTCAACAAGCCGTTAGTTTTGGCCACTGACTGACTAACGGCTTTGACTAATAG
C9ORF72-04b stem loop	CCTGCTATTAGTCAAAGCCGTTAGTCAGTCAGTGGCCAAAACTAACGGCTTGTTGACTAATAGC

Supplementary Table 2 - Proteomics analysis revealed significant differential expression of 24 proteins

LOG2 (Fold Change)	Identified Proteins	Gene name	p-value
-4.128008727	synaptic vesicle glycoprotein 2A	sv2a	0.02070914
-3.654641415	calpastatin	cast	0.0416028
-3.128015343	hydroxy-3-methylglutaryl-Coenzyme A synthase 1 (soluble) (AAH49456.1)	hmgcs1	0.02950878
-2.863542157	Alkaline phosphatase	alpl	0.03635185
-2.737198494	Methylenetetrahydrofolate dehydrogenase (NADP+ dependent)	mthfd2	0.02270433
-2.404288943	L-2-hydroxyglutarate dehydrogenase, mitochondrial	l2hgdh	0.04519278
-2.391186305	integrin alpha-2-like isoform X1	itga2.2	0.03836482
-2.246910845	adapter molecule crk	crk	0.04630673
-2.077591185	complement C5	c5	0.03424657
-2.054375027	Oxct1b protein	oxct1b	0.04522116
-1.810816378	dmX-like protein 2	dmxl2	0.04981243
-1.791570505	uncharacterized protein LOC550458 isoform 1 [Danio rerio]	tubb6	0.04704342
1.824214917	fast skeletal myosin light chain 3	mylz3	0.0492682
1.993865177	myosin, light chain 1, alkali; skeletal, fast	myl1	0.04369252
2.254058278	Apolipoprotein A-IV	apoa4b.1	0.0309075
2.28305222	cytochrome c oxidase subunit 7A2 like	cox7a2l	0.04688909
2.440741318	Enolase 3, (beta, muscle)	eno3	0.02544621
2.500419058	serine/threonine-protein phosphatase 2A catalytic subunit beta isoform	ppp2cb	0.04183156
2.538029927	uncharacterized protein si:dkey-9l20.3	si:dkey-9l20.3	0.0401815
2.662770409	uncharacterized protein LOC768128	cst14b.1	0.02160492
2.986970782	nonhistone chromosomal protein HMG-14A-like	hmgn7	0.0220809
3.182978967	aspartate--tRNA ligase, cytoplasmic	dars1	0.03100769
3.239143855	Cluster of Zgc:112374 [Danio rerio] (AAI64397.1)	pon3.1	0.04934178
3.709872848	parvalbumin 8	pvalb8	0.0393578
