# Supplementary material for: Reduced C9orf72 function leads to defective synaptic vesicle release and neuromuscular dysfunction in zebrafish
Source: Commun Biol. 2021 Jun 25;4:792. doi: 10.1038/s42003-021-02302-y (PMC8233344; doi:10.1038/s42003-021-02302-y)
Supplement: Supplementary file 3 — Description of Additional Supplementary Files [file 42003_2021_2302_MOESM3_ESM.pdf]

Description of Additional Supplementary Files

File name: Supplementary Movie 1

Description: Free swimming adult control fish.

File name: Supplementary Movie 2

Description: Free swimming adult C9-miR fish.

File name: Supplementary Data 1

Description: The source data behind the graphs in the paper.
