# Supplementary material for: Reduced C9orf72 function leads to defective synaptic vesicle release and neuromuscular dysfunction in zebrafish
Source: Commun Biol. 2021 Jun 25;4:792. doi: 10.1038/s42003-021-02302-y (PMC8233344; doi:10.1038/s42003-021-02302-y)
Supplement: Supplementary file 7 — Reporting Summary [file 42003_2021_2302_MOESM7_ESM.pdf]

nature research

Corresponding author(s): Patten, Shunmoogum (Kessen); COMMSBIO-20-2120B

Last updated by author(s): May 6, 2021

Reporting Summary

Nature Research wishes to improve the reproducibility of the work that we publish. This form provides structure for consistency and transparency in reporting. For further information on Nature Research policies, see our Editorial Policies and the Editorial Policy Checklist.

Statistics

For all statistical analyses, confirm that the following items are present in the figure legend, table legend, main text, or Methods section.

n/a	Confirmed	
☐	☒	The exact sample size (*n*) for each experimental group/condition, given as a discrete number and unit of measurement
☐	☒	A statement on whether measurements were taken from distinct samples or whether the same sample was measured repeatedly
☐	☒	The statistical test(s) used AND whether they are one- or two-sided<br>*Only common tests should be described solely by name; describe more complex techniques in the Methods section.*
☒	☐	A description of all covariates tested
☒	☐	A description of any assumptions or corrections, such as tests of normality and adjustment for multiple comparisons
☐	☒	A full description of the statistical parameters including central tendency (e.g. means) or other basic estimates (e.g. regression coefficient) AND variation (e.g. standard deviation) or associated estimates of uncertainty (e.g. confidence intervals)
☐	☒	For null hypothesis testing, the test statistic (e.g. *F*, *t*, *r*) with confidence intervals, effect sizes, degrees of freedom and *P* value noted<br>*Give P values as exact values whenever suitable.*
☒	☐	For Bayesian analysis, information on the choice of priors and Markov chain Monte Carlo settings
☒	☐	For hierarchical and complex designs, identification of the appropriate level for tests and full reporting of outcomes
☒	☐	Estimates of effect sizes (e.g. Cohen's *d*, Pearson's *r*), indicating how they were calculated

Our web collection on statistics for biologists contains articles on many of the points above.

Software and code

Policy information about availability of computer code

Data collection	Locomotor activity was automatically recorded using the DanioVision recording chamber (Noldus). Confocal images were acquired using a Zeiss microscope (LSM780). Electrophysiological data were acquired using the Axopatch 200B and pCLAMP10 software (Molecular Devices). Proteome Discover (Version 2.3) developed by Thermo Scientific was used in the LCMS workflow for peptide identification.
Data analysis	Behavioural analysis was performed using the Ethovision XT 12 software (Noldus) to quantify the distance swam in zebrafish larvae. Electrophysiological data analyses were performed using the pCLAMP10 software. ImageJ manual tracking plugin software was used to quantify distance moved by adult zebrafish. ImageJ and Zeiss Zen software were used to quantify observations of images. Protein database searching was performed with Mascot 2.6 (Matrix Science) against the Refseq Danio Rerio (Zebrafish) protein database. Proteomic analysis was performed using Scaffold (Version 4.8). All statistical analyses were performed using the Graphpad PRISM software 8.

For manuscripts utilizing custom algorithms or software that are central to the research but not yet described in published literature, software must be made available to editors and reviewers. We strongly encourage code deposition in a community repository (e.g. GitHub). See the Nature Research guidelines for submitting code & software for further information.

Data

Policy information about availability of data

All manuscripts must include a data availability statement. This statement should provide the following information, where applicable:

- Accession codes, unique identifiers, or web links for publicly available datasets
- A list of figures that have associated raw data
- A description of any restrictions on data availability

The data as well as the material used in this study will be available on request. Mass spectrometry data generated in this study has been deposited in the ProteomeXchange repository and the accession code is provided in the Data availability section of the manuscript.

Field-specific reporting

Please select the one below that is the best fit for your research. If you are not sure, read the appropriate sections before making your selection.

[x] Life sciences [ ] Behavioural & social sciences [ ] Ecological, evolutionary & environmental sciences

For a reference copy of the document with all sections, see nature.com/documents/nr-reporting-summary-flat.pdf

Life sciences study design

All studies must disclose on these points even when the disclosure is negative.

Sample size	Sample size was determined based on previous experience with similar behavioural, imaging and electrophysiology experiments.
Data exclusions	No data were excluded from the analyses.
Replication	All fluorescent imaging, behavioural analyses, immunoblot data, qPCR analyses and electrophysiological analyses were successfully replicated and representative results were shown.
Randomization	All samples and animals for the experiments were randomly selected.
Blinding	Blinding was not performed prior to experimentation, as we needed to select transgenic animals using the gfp crystallin promoter to ensure insertion of C9-miRNA.

Reporting for specific materials, systems and methods

We require information from authors about some types of materials, experimental systems and methods used in many studies. Here, indicate whether each material, system or method listed is relevant to your study. If you are not sure if a list item applies to your research, read the appropriate section before selecting a response.

Materials & experimental systems

n/a	Involved in the study	
[ ]	[x]	Antibodies
[x]	[ ]	Eukaryotic cell lines
[x]	[ ]	Palaeontology and archaeology
[ ]	[x]	Animals and other organisms
[x]	[ ]	Human research participants
[x]	[ ]	Clinical data
[x]	[ ]	Dual use research of concern

Methods

n/a	Involved in the study	
[x]	[ ]	ChIP-seq
[x]	[ ]	Flow cytometry
[x]	[ ]	MRI-based neuroimaging

Antibodies

Antibodies used

1. Tetramethylrhodamine α-bungarotoxin- Thermofisher, T1175
2. Anti-SV2a- Developmental Studies Hybridoma Bank, SV2; AB_2315387
3. Anti-Rab3a - Sigma-Aldrich, WH0005864M1
4. Alexa Fluor 488-conjugated secondary antibody: Life Technologies, R37120
5. Anti- C9orf72: Novus Npb2
6. Anti- β-actin: Sigma-Aldrich, A5441
7. Anti- Choline Acetyltransferase, Sigma-Aldrich, AB144P
8. Anti-TDP-43:Proteintech, 10782-2-AP
9. Anti- C9orf72: Abcam, ab221137 (HEK cells experiments)
10. Anti-Myc: Sigma-Aldrich, M5546

Validation	1. https://www.thermofisher.com/order/catalog/product/T1175#/T1175<br>2. https://dshb.biology.uiowa.edu/SV2<br>3. https://www.sigmaaldrich.com/catalog/product/sigma/wh0005864m1?lang=en&region=CA<br>4. https://www.thermofisher.com/antibody/product/Goat-anti-Mouse-IgG-H-L-Cross-Adsorbed-Secondary-Antibody-Polyclonal/R37120<br>5. https://www.novusbio.com/products/c9orf72-antibody_nbp2-15656<br>6. https://www.sigmaaldrich.com/catalog/product/sigma/a5441?lang=en&region=CA<br>7. https://www.sigmaaldrich.com/catalog/product/mm/ab144p?lang=en&region=CA<br>8. https://www.ptglab.com/products/TARDBP-Antibody-10782-2-AP.htm<br>9.https://www.abcam.com/c9orf72-antibody-epr22021-ab221137.html<br>10. https://www.sigmaaldrich.com/catalog/product/sigma/m5546?lang=en&region=CA

Animals and other organisms

Policy information about studies involving animals; ARRIVE guidelines recommended for reporting animal research

Laboratory animals	Zebrafish (Danio rerio) was used as model organism. Both sexes were used and larvae were used from them.
Wild animals	This study did not involve wild animals.
Field-collected samples	This study did not involve samples collected from the field.
Ethics oversight	This study was carried out in accordance with the guidelines of the Canadian Council for Animal Care and received approval from the INRS-CNBE (Canada) ethics committee.

Note that full information on the approval of the study protocol must also be provided in the manuscript.
